# CCR2 Mediates Chronic LPS-Induced Pulmonary Inflammation and Hypoalveolarization in a Murine Model of Bronchopulmonary Dysplasia

**DOI:** 10.3389/fimmu.2020.579628

**Published:** 2020-10-06

**Authors:** Tracy X. Cui, Alexander E. Brady, Christina T. Fulton, Ying-Jian Zhang, Liza M. Rosenbloom, Adam M. Goldsmith, Bethany B. Moore, Antonia P. Popova

**Affiliations:** ^1^Division of Pediatric Pulmonology, Department of Pediatrics, University of Michigan Medical School, Ann Arbor, MI, United States; ^2^Division of Pulmonary and Critical Care Medicine, Department of Internal Medicine, University of Michigan Medical School, Ann Arbor, MI, United States; ^3^Department of Microbiology and Immunology, University of Michigan, Ann Arbor, MI, United States

**Keywords:** prematurity, bronchopulmonary dysplasia, inflammation, macrophages, CCR2

## Abstract

The histopathology of bronchopulmonary dysplasia (BPD) includes hypoalveolarization and interstitial thickening due to abnormal myofibroblast accumulation. Chorioamnionitis and sepsis are major risk factors for BPD development. The cellular mechanisms leading to these lung structural abnormalities are poorly understood. We used an animal model with repeated lipopolysaccharide (LPS) administration into the airways of immature mice to simulate prolonged airway exposure to gram-negative bacteria, focusing on the role of C-C chemokine receptor type 2-positive (CCR2+) exudative macrophages (ExMf). Repetitive LPS exposure of immature mice induced persistent hypoalveolarization observed at 4 and 18 days after the last LPS administration. LPS upregulated the expression of lung pro-inflammatory cytokines (TNF-*α*, IL-17a, IL-6, IL-1*β*) and chemokines (CCL2, CCL7, CXCL1, and CXCL2), while the expression of genes involved in lung alveolar and mesenchymal cell development (PDGFR-*α*, FGF7, FGF10, and SPRY1) was decreased. LPS induced recruitment of ExMf, including CCR2+ ExMf, as well as other myeloid cells like DCs and neutrophils. Lungs of LPS-exposed CCR2−/− mice showed preserved alveolar structure and normal patterns of *α*-actin and PDGFR*α* expression at the tips of the secondary alveolar crests. Compared to wild type mice, a significantly lower number of ExMf, including TNF-*α*+ ExMf were recruited to the lungs of CCR2−/− mice following repetitive LPS exposure. Further, pharmacological inhibition of TLR4 with TAK-242 also blocked the effect of LPS on alveolarization, *α*-SMA and PDGFR*α* expression. TNF-*α* and IL-17a induced *α*-smooth muscle actin expression in the distal airspaces of E16 fetal mouse lung explants. In human preterm lung mesenchymal stromal cells, TNF-*α* reduced mRNA and protein expression of PDGFR-*α* and decreased mRNA expression of WNT2, FOXF2, and SPRY1. Collectively, our findings demonstrate that in immature mice repetitive LPS exposure, through TLR4 signaling increases lung inflammation and impairs lung alveolar growth in a CCR2-dependent manner.

## Introduction

Bronchopulmonary dysplasia (BPD) is the most common chronic pulmonary complication of preterm birth, with over 12,000 new cases annually in the US (1). Infants with BPD have higher complication rates, longer initial hospitalizations (2), and often develop chronic respiratory symptoms and abnormal lung function with airflow obstruction lasting into adulthood (3). Despite its impact on chronic respiratory disease, few measures to prevent BPD exist and treatment is supportive rather than curative.

The histopathology of BPD includes hypoalveolarization and interstitial thickening due to abnormal myofibroblast accumulation (4). The mechanisms leading to these lung structural abnormalities are poorly understood. Infection and inflammation have been postulated to play a role in impaired alveolar growth in BPD [reviewed in (5, 6)]. There is ample evidence that chorioamnionitis and sepsis are major risk factors for BPD development (7–9). In severe chorioamnionitis, amniotic fluid pro-inflammatory cytokine levels correlate with BPD severity (10, 11). Furthermore, postnatal airway gram-negative bacterial dominance is associated with BPD development (12–14). In premature infants undergoing prolonged (more than 2 weeks) mechanical ventilation, neonatal airway colonization with gram negative, but not gram positive bacteria is associated with development of severe BPD and prolonged use of supplemental oxygen (15, 16). During the first days of life, an influx of polymorphonuclear leukocytes and macrophages is observed in tracheal aspirates of mechanically ventilated premature infants who later develop BPD (17, 18). Monocytes from tracheal aspirates of premature infants with evolving BPD display a pro-inflammatory phenotype and increased expression of IL-1 cytokines (19). Activated alveolar macrophages producing oxidants are present in the airways of infants with established BPD (20). Tracheal aspirate levels of pro-inflammatory cytokines such as IL-6, IL-1*β*, TNF-*α*, and chemokines CCL2, CCL7, CCL8, and IL-8 (CXCL8) are higher in premature infants with respiratory distress syndrome who develop BPD (21–26). Similar findings are present in animal models of BPD (27–29).

Bacterial endotoxin lipopolysaccharide (LPS) is a membrane component of Gram-negative bacterial wall that binds to Toll-like receptor 4 (TLR4), as well as CD14, the macrophage scavenger receptor, the *β*2 integrins (CD11a/CD18, CD11b/CD18, and CD11c/CD18) and caspase 11 (30–32). LPS is capable of inducing a strong pro-inflammatory immune response, including increased CCL2 expression (33). CCL2 is a high-affinity ligand for CC chemokine receptor 2 (CCR2). CCR2 mediates recruitment and accumulation of monocytes, dendritic cells and exudative macrophages in response to various infectious stimuli (34–36). CCR2 signaling is required for lung pro-fibrotic responses in mice (37–39) and partially mediates hyperoxia-induced hypoalveolarization in immature mice (40). Recent studies implicate a role for repetitive LPS exposure in pulmonary hypoalveolarization and inflammation, including increased CCL2 expression (13, 41–43). However, the role of innate immune cells recruited in response to increased CCL2 expression and their pro-inflammatory signals has not been investigated. In this study, we tested the hypothesis that in immature mice repetitive LPS exposure, through TLR4 signaling increases lung inflammation and impairs lung alveolar growth in a CCR2-dependent manner.

## Materials and Methods

### Ethics Statement

The animal study was performed in strict accordance with the NIH Guide for the Care and Use of Laboratory Animals recommendations. The protocol was approved by the University of Michigan Committee on Use and Care of Animals.

### Animal Model

Neonatal C57BL/6J and CCR2−/− (B6.129S4-*Ccr2^tm1Ifc^*/J) mice from Jackson Laboratories (Bar Harbor, ME) were inoculated with 3 µg/10 µl of LPS from *E. coli* O26:B6 (Sigma-Aldrich, St. Louis, MO), or endotoxin-free PBS (Sigma-Aldrich), intranasally on days of life 3, 5, 7, and 10. Selected mice were injected intraperitoneally with 10 mg/kg of the TLR4 inhibitor TAK-242 (MilliporeSigma, Burlington, MA) or dimethyl sulfoxide control (Sigma-Aldrich) dissolved in 40 µl of endotoxin-free PBS per mouse.

### Quantitative Real-Time PCR

Whole lung tissues were harvested in TRIzol (Zymo Research, Irvine, CA) and homogenized on ice. RNA extraction was performed according to Direct-zol™ RNA MiniPrep (Zymo Research, Irvine, CA) manufacturer instructions. Complementary DNA was synthesized following the TaqMan Reverse Transcription Reagents protocol (Life Technologies, Carlsbad, CA). The expression levels of genes of interest were quantified with SYBR Green technology. The specific primer sequences are available upon request. Relative gene expression was analyzed with the 2−ΔCT algorithm by normalizing the level of gene expression for each sample to glyceraldehyde 3-phosphate dehydrogenase (GAPDH) and beta-actin (*β*-actin) as indicated.

### Measurement of Lung Cytokines

Protein levels of TNF-*α*, IL-6, and IL-17a in whole lung homogenate supernatants were measured by enzyme-linked immunosorbent assay (ELISA). Tracheal aspirate protein levels of TNF-*α* were measured by multiplex assay (Bio-Rad, Hercules, CA).

### Flow Cytometry Analysis

Lungs were perfused with PBS containing EDTA (0.5 mM), minced, and digested with Liberase TM (100 µg/ml; Roche, Indianapolis, IN), together with collagenase XI (250 µg/ml), hyaluronidase 1a (1 mg/ml), and DNase I (200 µg/ml; Sigma, St. Louis, MO) for 1 h at room temperature (44). Dead cells were stained with Pac-Orange Live/Dead fixable, dead staining dye (Invitrogen). Cells were filtered through a 70 µm strainer, treated with RBC lysis buffer (BD Biosciences, Franklin Lakes NJ), and kept on ice in media containing 10% serum. Lung cells were then stained with fluorescently labeled antibodies against various leukocyte surface markers (CD45, CD11b, CD11c, F4/80, and CCR2). Appropriate isotype-matched controls and Fluorescence Minus One (FMO) controls were used in all experiments. Antibodies were purchased from EBiosciences (San Diego, CA) or Biolegend (San Diego, CA). Cells were fixed and analyzed on a Fortessa (Becton-Dickinson, San Jose, CA) or FACSAria II (BD Biosciences) flow cytometer. Results were analyzed using FlowJo software (Tree Star, Ashland, OR).

### Histology, Morphometry and Immunofluorescence Microscopy

Lungs were perfused with 5 mM EDTA, inflated to 30 cmH2O pressure with 4% paraformaldehyde (Sigma-Aldrich, St. Louis, MO), and paraffin embedded. Total lung volume was determined by Scherle’s method based on the total water displacement (45, 46). Five μm-thick paraffin sections were stained with Hematoxylin and eosin (H&E) or fluorescently labeled, monoclonal antibodies specific for PDGFR-*α* (Cell Signaling, Danvers, MA) or *α*-smooth muscle actin (Sigma-Aldrich). Images were acquired using Olympus IX71 microscope (Tokyo, Japan). To assess alveolarization, alveolar chord length was determined as described (47). In summary, alveolar chord length was calculated as the mean length of line segments on random test lines spanning the airspace between intersections of the line with the alveolar surface was calculated. Four random images from two sections for each animal were photographed at ×200.

### Human Tracheal Aspirate Collection and MSC Isolation

The human study was approved by the University of Michigan Medical School Institutional Review Board. Written informed consent was obtained from both parents. All methods involving human participants were performed in accordance with the relevant guidelines and regulations. Tracheal aspirates from premature infants (≤32 weeks gestation), requiring mechanical ventilation for respiratory distress syndrome in the first 7 days of life, were collected during routine suctioning. Primary preterm lung MSCs were isolated from the tracheal aspirates as previously described (48). Supernatants were assayed by multiplex assay (Millipore) for CCL2 and TNF-*α*.

### Human Tracheal Aspirate MSC Cell Culture and RNA Isolation

Preterm lung MSCs were maintained in 10% MSC fetal bovine serum, 1% penicillin–streptomycin, 1% L-glutamine, and 0.5% amphotericin B in a 100-mm plate, at 37°C and 5% CO2, until 80–90% confluent. Cells were then treated with recombinant human TNF-*α* (5 ng/ml). The cells were serum starved for 4 h prior to harvest to minimize differences in cell cycle related gene expression. Total RNA was extracted using RNeasy Plus Mini kit (Qiagen, Valencia, CA). Gene expression was analyzed by RT-qPCR or immunoblotting.

### Immunoblotting

Lysates were resolved by SDS-PAGE and transferred to a nitrocellulose membrane. Membranes were blocked in 5% milk for 1 h at room temperature and probed with antibodies against PDGFR-*α* (Cell Signaling, Danvers, MA).

### Lung Explant Culture

Embryonic day 16 fetal mouse lungs were minced into 0.5–1.0 mm^3^ pieces. Explants were placed onto polyethylene hanging insert filters (0.4 μm, EMD Millipore Corp, Billerica, MA) in 12-well plates and were cultured in the presence or absence of recombinant mouse TNF-*α* (50 ng/ml) and IL-17a (100 ng/ml), both from Peprotech, (Rocky Hill, NJ), for 72 h. Total RNA was extracted using RNeasy Plus Micro kit (Qiagen, Valencia, CA). Gene expression was analyzed by RT-qPCR. Selected explants were fixed in 4% paraformaldehyde overnight and stained with fluorescently labeled, monoclonal antibody specific for *α*-smooth muscle actin (Sigma-Aldrich).

### Statistical Analysis

All data were described as mean ± SE or median and interquartile range as appropriate. An unpaired *t*-test, Mann–Whitney test or one-way ANOVA test were used for comparison. *P* values were considered statistically significant if they were <0.05.

## Results

### Chronic Neonatal LPS Exposure Induces Persistent Hypoalveolarization

**Figure 1:**
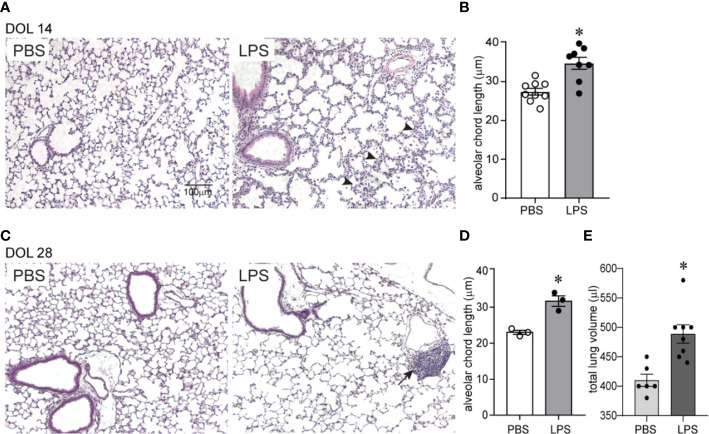
Chronic airway LPS exposure induces persistent hypoalveolarization in immature mice. Immature C57BL/6J mice were inoculated with 3 µg/10 µl of LPS from *E.coli* O26:B6 or equal volume endotoxin-free PBS intranasally on days of life 3, 5, 7, and 10. Lung histology was assessed on days of life 14 and 28. Representative lung sections were stained with hematoxylin and eosin. Compared to PBS-exposed mice, LPS-exposed mice demonstrated increased alveolar size indicative of alveolar simplification both on day of life 14 **(A)** and 28 **(C)**. Hypoalveolarization in LPS-exposed mice was associated with a statistically significant increase in alveolar chord length both on day of life 14 **(B)** and 28 **(D)**. N = 8–9 per group for day of life 14 and three per group for day of life 28, mean ± SEM, *different from PBS-exposed group, *P < 0.05, unpaired *t*-test. LPS-exposed lungs also showed leukocyte infiltration within the interstitium and in the alveolar spaces (arrowheads in **A**) on day of life 14 and collection of mononuclear cells, resembling BALT, in the perivascular space (arrows in **C**) on day of life 28. **(E)** On day of life 28, the total lung volume of LPS-treated lungs was significantly higher compared to PBS-treated lungs. *different from PBS-exposed group, *P < 0.01, unpaired *t*-test.

We examined the effects of chronic airway LPS exposure in early life on lung alveolar development. Immature mice were anesthetized and repeatedly inoculated with LPS or endotoxin-free PBS intranasally on days of life 3, 5, 7, and 10. Subsequently, we examined lung histology on days of life 14 and 28. Comparative examination of random H&E images revealed that following LPS exposure: alveolar chord length was significantly increased both on days of life 14 and 28, indicating long lasting interference with lung alveolar growth (**Figure 1**). In addition, LPS-exposed lungs showed leukocyte infiltration within the interstitium and alveolar spaces (**Figure 1** arrowheads) on day of life 14. Collections of mononuclear cells localized around the bronchovascular bundles or perivascular space, resembling BALT, were observed on day of life 28 (**Figure 1** arrows). In addition, on day of life 28, the total lung volume of LPS-treated lungs was significantly higher compared to PBS-treated lungs suggestive of hyperinflation (**Figure 1E**).

### Chronic Airway LPS Exposure Induces Lung Pro-Inflammatory Cytokine and Chemokine Expression and Downregulates the Expression of Genes Involved in Lung Alveolar and Mesenchymal Cell Development

**Figure 2:**
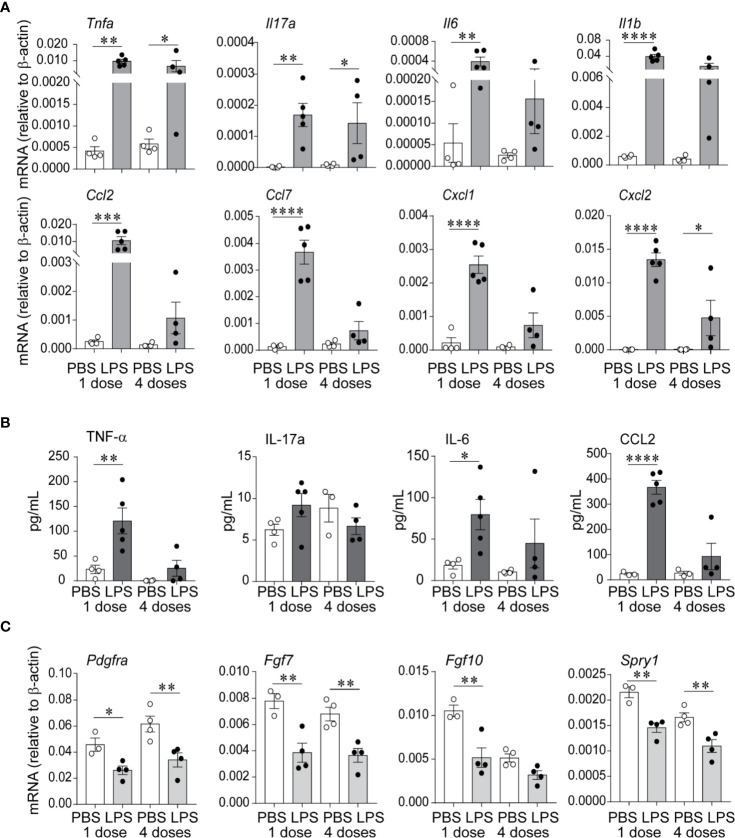
Repetitive airway LPS exposure induces lung pro-inflammatory cytokine and chemokine expression and downregulates the expression of genes required for lung alveolar and mesenchymal cell development. Immature mice were inoculated with LPS or PBS intranasally on days of life 3, 5, 7, and 10. Whole lung mRNA and protein were measured by quantitative PCR and ELISA after the first and after the last (fourth) LPS inocculation. **(A)** LPS increased whole lung mRNA expression of *Tnfa, Il17a, Il6, Il1b, Ccl2, Ccl7, Cxcl1*, and *Cxcl2*. **(B)** Effects of LPS on whole lung protein expression of TNF-*α*, IL-17a, IL-6, and CCL2. **(C)** LPS decreased whole lung mRNA expression of *Pdgfra, Fgf7, Fgf10*, and *Spry1*. (n = 4–5 per group, mean ± SEM, *P < 0.05, **P < 0.01, ***P < 0.001, ****P < 0.0001, one-way ANOVA). The experiment was repeated three times with similar results.

We examined lung pro-inflammatory cytokine and chemokine expression in response to LPS, both after the first and after the fourth (repetitive) LPS administration. *Tnfa, Il17a, Il6*, and *Il1b* mRNA expression was significantly increased on day 1 after the first LPS inoculation (**Figure 2A**). The pattern of expression was similar on day 1 after repetitive LPS administration. On protein level, TNF-*α* and IL-6 levels were significantly increased after the first LPS inoculation and tended to be higher after repetitive LPS administration compared to PBS-treated mice (**Figure 2B**). In addition, mRNA and protein expression of CCL2, a potent monocyte chemoattractant, as well as mRNA expression of other chemokines like CCL7, CXCL1, and CXCL2 were also increased in a similar pattern (**Figures 2A, B**).

To define the effects of LPS on lung alveolar development, we focused on the expression of *Pdgfra, Fgf7, Fgf10*, and *Spry1*, mesenchymal cell genes required for lung alveolar growth and mesenchymal cell function (49–54). We found that both after the first and after repetitive LPS administration, the mRNA expression of *Pdgfra, Fgf7, Fgf10*, and *Spry1* was significantly decreased (**Figure 2C**), correlating with the impaired alveolar growth at this time point.

### Chronic Airway LPS Exposure Promotes Lung Myeloid Cell Infiltration

**Figure 3:**
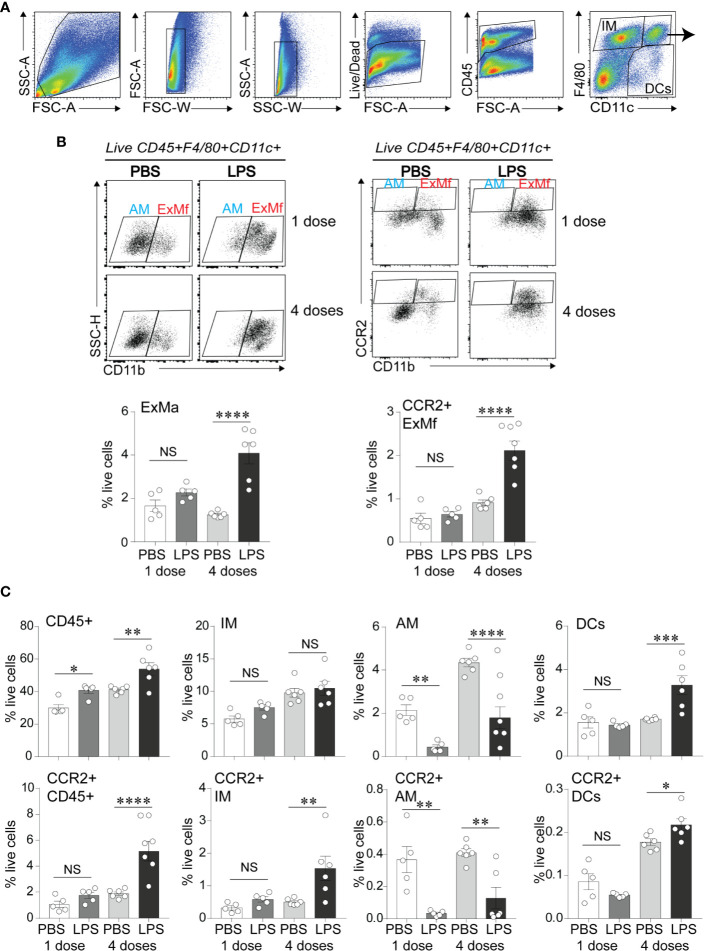
CCR2+ exudative macrophages are recruited to neonatal lungs after repetitive airway LPS exposure. Immature mice were inoculated with LPS or PBS intranasally on days of life 3, 5, 7, and 10. Lung digests obtained after the first and after the last (fourth) LPS inocculation were subjected to flow cytometry. **(A)** Flow gating strategy used to identify viable, CD45+ cell populations of lung macrophages (F4/80+CD11c+), dendritic cells (DCs; F4/80− CD11c+) and interstitial macrophages/monocytes (IMs; F4/80+CD11c−). **(B)** Lung F4/80+CD11c+ cells were analyzed for expression of CD11b to distinguish alveolar macrophages (AMs; F4/80+CD11c+CD11b−) from exudative macrophages (ExMf; F4/80+CD11c+CD11b+), and for expression of CCR2 to determine the CCR2-expressing AMs and ExMf, defined by the gates based on FMO control. The populations of ExMf and CCR2+ ExMf were significantly increased after repetitive LPS exposure. **(C)** Comparison of total live, CD45+ cells, IMs, AMs, DCs, as well as CCR2+CD45+, CCR2+IMs, CCR2+ AMs, CCR2+DCs in PBS- and LPS-exposed lungs. N = 3–6 mice per group. NS is non-significant difference, *P < 0.05, **P < 0.01, ***P < 0.001, ****P < 0.0001, one-way ANOVA.

To define the effects of LPS exposure on lung myeloid cell responses on day 1 after the first and after repetitive LPS administration, we examined lungs by flow cytometry. We differentiated the exudative macrophages (ExMf) from dendritic cells (DCs), alveolar macrophages (AMs), and interstitial macrophages (IMs) based on the established gating strategy using F4/80, CD11c and CD11b expression (27, 55) (**Figures 3A, B**). There was a significant increase in CD45-positive cells both after the first and after repetitive LPS administration (**Figure 3C**). The percent CD45-positive cells that expressed CCR2 was significantly higher after repetitive LPS inoculation (**Figure 3C**). A similar trend was noted after the first LPS inoculation. We focused on exudative macrophages, which migrate to the lungs following LPS inoculation in CCR2-dependent manner (34, 37). Exudative macrophages are increased in a hyperoxia-induced hypoalveolarization model of BPD (27). Comparison of the magnitude of increase in ExMf and CCR2-positive ExMf after the first and after repetitive LPS stimulation demonstrated a significantly greater increase in both after repetitive LPS exposure (**Figure 3B**). Similarly, repetitive LPS exposure induced an increase in DCs, including CCR2+ DCs, as well as CCR2+ interstitial macrophages (**Figure 3C**). We noticed that LPS decreased the AM population at both time points (**Figure 3C**). We identified neutrophils by using a series of gates (CD45+ F4/80− CD11c− CD11b+ SSC mod-high FSC high, **Supplementary Figure 1**). We observed that both after the first and after repetitive LPS stimulation there is a significant increase in neutrophils in the lungs (**Supplementary Figure 1**). CCR2-positive neutrophils were significantly increased after repetitive LPS exposure but constituted a significantly smaller proportion of all live cells compared to the CCR2+ ExMf. Together, these data show that in immature mice the lung myeloid cell response to a single dose of LPS is dominated by neutrophils, whereas the response to repetitive LPS stimulation includes ExMf and DCs, in addition to neutrophils. A significantly higher proportion of these cells, especially ExMf, express CCR2.

### Role of CCR2 in LPS-Induced Hypoalveolarization and LPS Effects on Interstitial α-Smooth Muscle Actin and PDGFR-*α* Expression in the Alveolar Tips

**Figure 4:**
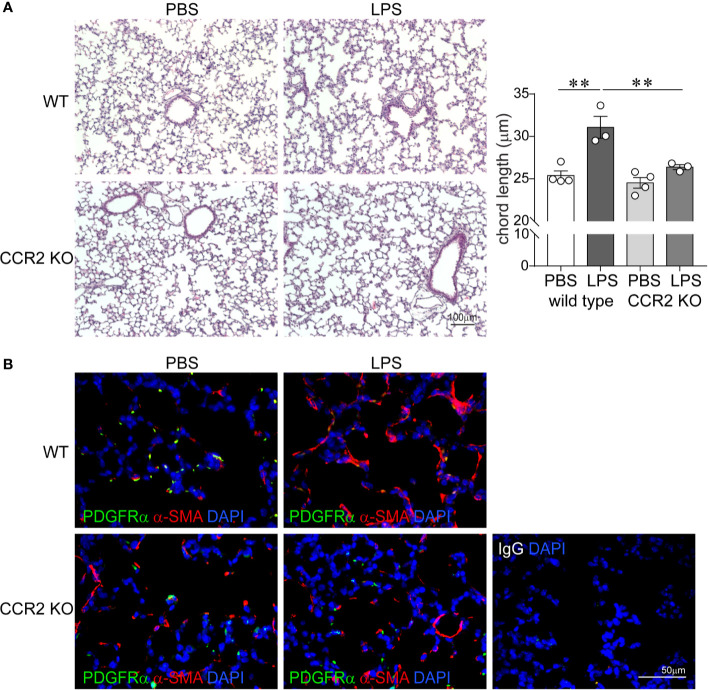
CCR2 deficiency attenuates repetitive LPS-induced hypoalveolarization and restores normal pattern of *α*-smooth muscle actin and PDGFR-*α* expression in the distal lung. Immature wild type and CCR2−/− mice were inoculated with LPS or PBS intranasally on days of life 3, 5, 7, and 10. Lung histology was assessed on day of life 14. **(A)** Representative lung sections were stained with hematoxylin and eosin. Unlike wild type mice, LPS-exposed CCR2−/− mice do not develop increased alveolar size indicative of alveolar simplification. After LPS exposure, the chord lengths of CCR2−/− mice were significantly lower compared to wild type mice. N = 3–4 per group, mean ± SEM, **different from PBS-exposed group, **P < 0.01. **(B)** Lung sections were stained for *α*-smooth muscle actin (red) and PDGFR-*α* (green). In wild type, PBS-treated mice, *α*-smooth muscle actin (red) and PDGFR-*α* (green) were predominantly at the tips of secondary crests. In contrast the wild type, LPS-exposed lung has abnormal *α*-smooth muscle actin (red) widespread throughout the interstitium, and there are very few PDGFR-*α* (green)-positive cells at the tips of secondary crests. In LPS-exposed CCL2−/− lungs *α*-smooth muscle actin (red) and PDGFR-*α* (green) were predominantly at the tips of secondary crests, a pattern similar to wild type mice. The representative images are from a random coronal section. The experiments were repeated two times with similar results.

To assess the effects of absent CCR2 signaling on chronic LPS-induced hypoalveolarization, we repeatedly inoculated immature wild type and CCR2−/− mice with LPS or endotoxin-free PBS intranasally on days of life 3, 5, 7, and 10. Subsequently, we examined lung histology on day of life 14. As noted above, in wild type mice chronic LPS exposure induced larger and fewer alveolar spaces resulting in increased alveolar chord lengths. In contrast, the alveolar size of LPS-exposed CCR2−/− mice appeared similar to PBS-treated CCR2−/− and wild type mice (**Figure 4A**). The chord lengths of LPS-exposed CCR2−/− mice were similar to the chord lengths of the PBS-treated mice and significantly smaller than the LPS-exposed wild type mice. Together, these data indicate that CCR2−/− mice are protected from the effects of LPS on alveolarization.

Next, we evaluated the effects of chronic LPS exposure on lung *α*-SMA and PDGFR-*α* expression in both wild type and CCR2−/− mice by using fluorescence microscopy. As lung saccules form, myofibroblasts that express PDGFR*α* and *α*-SMA migrate to the tips of secondary alveolar septa and are required for alveogenesis (49, 50, 56–58). By contrast, hyperoxia-exposed lungs with arrested alveolar development show a paucity of *α*-actin-positive myofibroblasts at the septal tips and an increase in the number of interstitial myofibroblasts (59). In BPD, alveolar septa are thickened with *α*-SMA-positive myofibroblasts (4). We have also previously reported that BPD lungs show a paucity of PDGFR*α*-positive mesenchymal cells in the dysmorphic alveolar septa (60). We inoculated immature wild type and CCR2−/− mice with LPS or PBS by intranasal inoculation on days of life 3, 5, 7, and 10. Subsequently, we examined lung histology on day of life 14. As previously described, in wild type PBS-treated lungs we observed *α*-SMA-positive cells predominantly at the tips of the secondary alveolar crests (**Figure 4B**), consistent with the location of alveolar myofibroblasts. These cells are also positive for PDGFR*α*. LPS exposure induced *α*-SMA expression in the interstitium and decreased PDGFR*α* immunoreactivity at the tips of the secondary alveolar crests (**Figure 4B**). In contrast, the lungs of LPS-treated CCR2−/− mice show a pattern similar to the wild type PBS-treated mice with *α*-SMA- and PDGFR*α*-positive cells predominantly at the tips of the secondary alveolar crests (**Figure 4B**). Collectively, these results demonstrate that chronic LPS-induced hypoalveolarization, increased interstitial *α*-SMA expression and reduced PDGFR*α* expression at the tips of the secondary alveolar crests are CCR2 dependent.

### CCR2 Is Required for Exudative Macrophage Infiltration and Increased TNF-*α* Expression in Response to Chronic LPS Exposure

**Figure 5:**
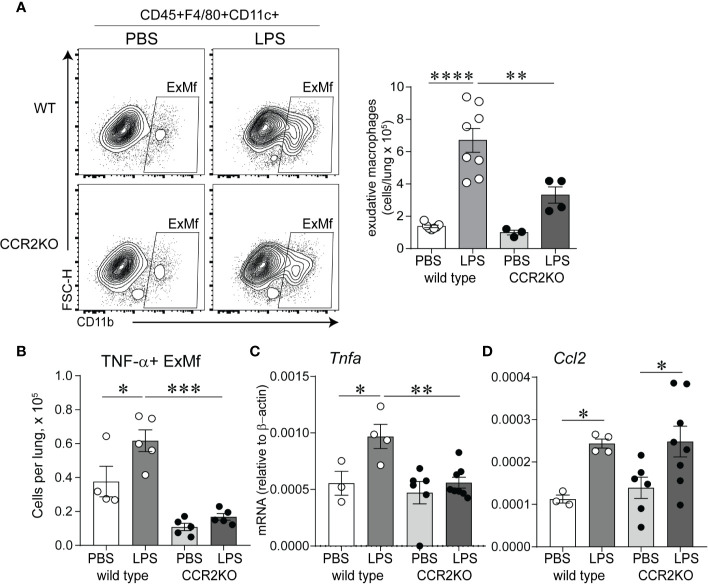
Immature CCR2 knockout mice show an attenuated increase in lung exudative macrophages and lung TNF-*α* expression following chronic LPS exposure. **(A)** CD45+F4/80+CD11c+CD11b+ cells, representing exudative macrophages (ExMf) were quantified by flow cytometry. Compared with LPS-exposed wild type mice, CCR2 knockout mice showed a decreased number of ExMf **(A)**, in addition to decreased number of TNF-*α*+ExMf **(B)** and total lung TNF-*α* mRNA expression **(C)**. LPS-induced Ccl2 mRNA expression was not affected by CCR2 deficiency **(D)**. n = 3 to 8 mice per group, *P < 0.05, **P < 0.01, ***P < 0.001, ****P < 0.0001, one-way ANOVA.

We hypothesized that CCR2 signaling is required for chronic LPS exposure-induced infiltration of the lungs with ExMf. To test this hypothesis, we inoculated immature wild type and CCR2−/− mice with LPS or PBS intranasally as above. We quantified lung ExMf using flow cytometry. As described above, in wild type mice LPS induced a significant increase in ExMf. In contrast, CCR2−/− mice showed an attenuated increase in ExMf (**Figure 5A**). The number of ExMf that were recruited to the lungs in response to repetitive LPS stimulation was significantly lower in CCR2−/− mice compared to wild type mice. We also analyzed the effect of the absence of CCR2 on LPS-induced recruitment of neutrophils. Neutrophils were distinguished from other myeloid cells in the lung based on gating for CD45+, Live, SSC hi, CD11b+, CD11c−, Gr-1+ (**Supplementary Figure 2**). As noted above, LPS induced a significant increase in the number of neutrophils in the lungs of wild type mice. In CCR2−/− mice, there was also a trend for an increase in neutrophils following LPS inoculation (**Supplementary Figure 2**). There was no significant difference in the number of neutrophils in wild type and CCR2−/− mice both at baseline and after repeated LPS inoculation. We investigated whether lung ExMf from wild type and CCR2−/− mice produce TNF-*α* after repetitive LPS exposure. The number of lung ExMf producing TNF-*α* after LPS exposure was significantly increased in wild type mice, whereas it remained low and unchanged in CCR2−/− mice (**Figure 5B**). Whole lung TNF-*α* mRNA expression was increased in wild type mice but did not change in CCR2−/− mice (**Figure 5C**). Unlike TNF-*α*, the effect of LPS on the expression of Ccl2 was not affected in CCR2−/− mice (**Figure 5D**), suggesting a different source for Ccl2. Together, these results highlight an important role for ExMf and TNF-*α* in chronic LPS-induced pulmonary inflammation in immature mice.

### Effects of Pharmacological Inhibition of TLR4 With TAK-242 on Chronic LPS-Induced Hypoalveolarization, Myofibroblast Differentiation, and Suppression of PDGFR-*α* Signaling

**Figure 6:**
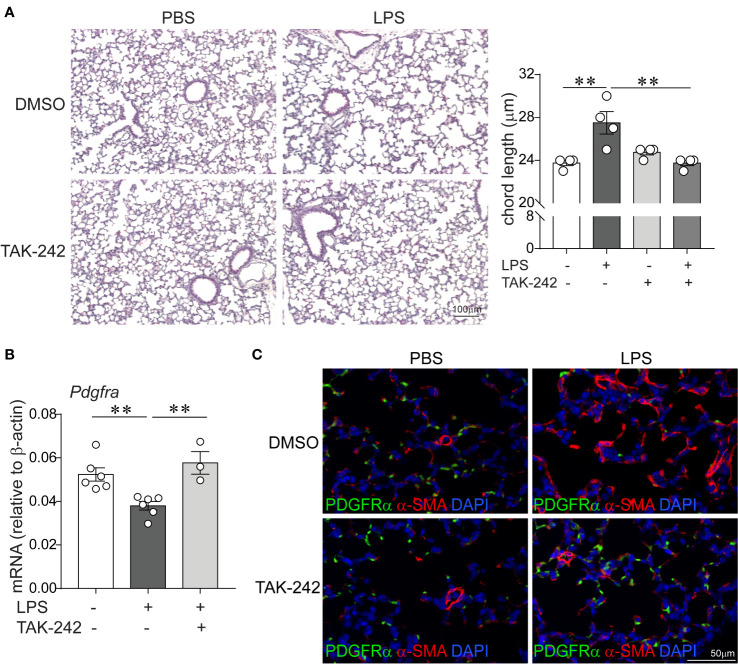
Pharmacological inhibition of TLR4 with TAK-242 attenuates the effects of chronic LPS exposure on alveolarization, *α*-smooth muscle actin and PDGFR-*α* expression in the distal lung. Immature wild type mice were inoculated intranasally with LPS or PBS and treated intraperitoneally with control (DMSO) or the TLR4 inhibitor TAK-242 on days of life 3, 5, 7, and 10. Lung histology was assessed on day of life 14. **(A)** Representative lung sections were stained with hematoxylin and eosin. Unlike control (DMSO)-treated mice, LPS-exposed TAK-242-treated mice do not develop increased alveolar size indicative of hypoalveolarization. After LPS exposure, the chord lengths of TAK-242-treated mice were significantly lower compared to control (DMSO)-treated mice. N = 3–4 per group, mean ± SEM, **different from PBS-exposed group, **P < 0.01. **(B)** Whole lung PDGFR-*α* mRNA expression was decreased after repetitive LPS exposed in control (DMSO)-treated, but not in TAK-242-treated mice. N = 3–6 per group, mean ± SEM, **different from PBS-exposed group, **P < 0.01. **(C)** Fluorescence microscopy shows after LPS exposure there is increased deposition of *α*-smooth muscle actin (red) in the alveolar interstitium and reeduced PDGFR-*α* expression (green) at the tips of secondary crests in control (DMSO)-treated mice, but not in TAK-242-treated mice. The representative images are from a random coronal section. The experiments were repeated two times with similar results.

Since LPS signals through binding and activation of TLR4, as well as other receptors including CD14, the macrophage scavenger receptor, the *β*2 integrins and caspase 11 (30–32), we investigated the requirement of TLR4 for the LPS effects on lung alveolar growth. Specifically, we tested the potential of the small molecule inhibitor of TLR4 TAK-242 (61) to block the effects of LPS on alveolarization and the expression of *α*-smooth muscle actin and PDGFR-*α* in the distal developing lung. Immature wild type mice were anesthetized and inoculated with LPS or PBS by intranasal inoculation on days of life 3, 5, 7, and 10, as described above. Immediately prior to each LPS or PBS inoculation, mice were injected intraperitoneally with TAK-242 or DMSO control. Subsequently, we examined lung histology on day of life 14. Compared to PBS-treated mice, the LPS-treated mice that were injected with the control DMSO showed larger and fewer alveoli and increased chord lengths (**Figure 6A**), consistent with impaired alveolar development. In contrast, alveolar size and chord lengths of the LPS-treated mice that were injected with TAK-242 appeared similar to the PBS-treated mice (**Figure 6A**). TAK-242 also blocked the decrease in PDGFR*α* mRNA and protein expression and prevented the increase in interstitial *α*-SMA induced by LPS (**Figure 6B**). These data indicate that TLR4 is required for the LPS effects on alveolarization, interstitial myofibroblast differentiation, represented by the *α*-SMA-positive cells and the suppression of PDGFR-*α* signaling.

### In E16 Fetal Mouse Lung Explants, TNF-*α* and IL-17a Induce *α*-Smooth Muscle Actin Expression in the Distal Airspaces

**Figure 7:**
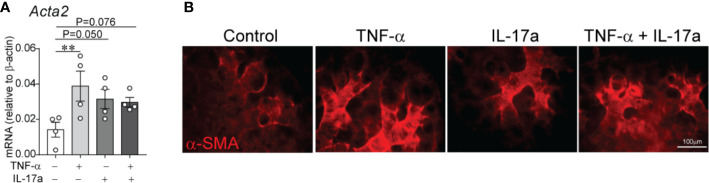
In E16 fetal mouse lung explants, TNF-*α* and IL-17a induce *α*-smooth muscle actin expression in the distal airspaces. E16 fetal mouse lung explants were microdissected and cultured *in vitro* in a transwell system on air–liquid interface in the presence or absence of recombinant mouse TNF-*α* (50 ng/ml), IL-17a (100 ng/ml) or both for 72 h. Control explants were cultured in serum free medium. This period corresponds to early saccular stage of lung development. Subsequently, lung explant tissue was analyzed for *α*-smooth muscle actin expression by qPCR **(A)** and immunostaining **(B)**. **(A)** Data are shown as mean ± SEM; **P < 0.01 by unpaired *t*-test.

At P3 the immature mouse lung is in late saccular stage of lung development that corresponds to late preterm human lung. To extend our study to encompass earlier stage of lung development as observed in extremely premature infants, we used E16 fetal lung explants corresponding to early saccular stage of development. To gain additional insight into the effects of LPS-induced pro-inflammatory signals on distal lung *α*-SMA expression, we investigated the effects of TNF-*α* and IL-17a, pro-inflammatory cytokines shown above to be induced in the lungs of immature mice exposed to LPS. To test this effect, we used an *in vitro* model in which E16 fetal mouse lungs were cultured on air–liquid interface for 72 h in the presence or absence of TNF-*α*, IL-17a or both. In a similar model, LPS has previously been shown to induce thick bands of *α*-SMA-positive myofibroblasts in the lung explant mesenchyme (62). We found that incubation with mouse recombinant TNF-*α* and IL-17a each and in combination induced *α*-SMA mRNA (**Figure 7A**) and protein (**Figure 7B**) expression in the distal airways of the lung explants. Our results, however, do not indicate an additive or synergistic effect between TNF-*α* and IL-17a. To some degree the combined treatment weakened the effect of each factor, which could be due to competing effects. TNF-*α* and IL-17a did not significantly affect the mRNA expression of PDGFR-*α*. Since PDGFR-*α* is required for alveolar stage of lung development and the E16 fetal lung explants are in early saccular stage of lung development, we did not focus on PDGFR-*α* in this model.

### Effect of TNF-*α* on Human Preterm Lung Mesenchymal Stromal Cell Expression of PDGFR-*α*

**Figure 8:**
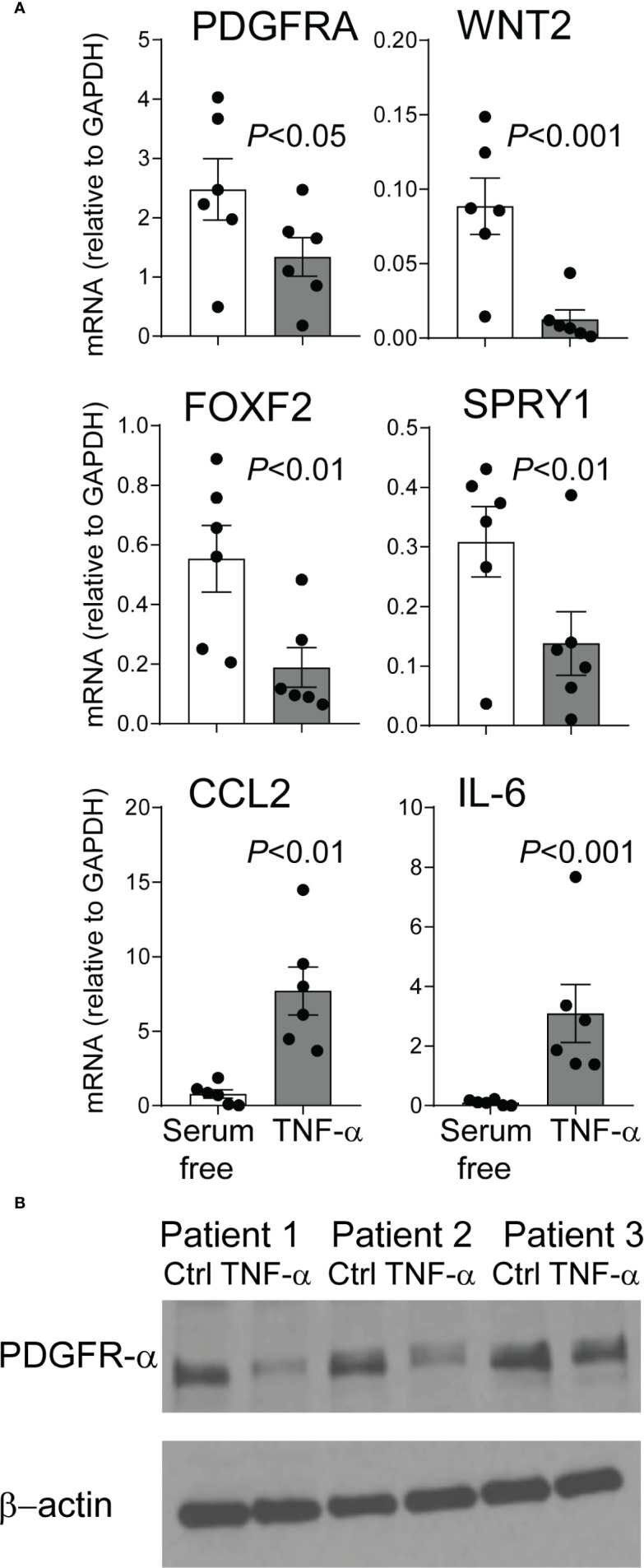
TNF-*α* treatment of mesenchymal stromal cells reduces PDGFR-*α* expression. Human preterm lung mesehncymal stromal cells (MSCs) were cultured in serum free medium with or without 5 ng/ml TNF-*α* for 48 h. PDGFR-*α* mRNA and protein expression was assessed by qPCR and ELISA, respectively. Also assessed was the mRNA expression of WNT2, FOXF2, SPRY1, CCL2, and IL-6 mRNA expression. **(A)** TNF-*α* treatment significantly reduced mRNA expression of PDGFRA, WNT2, FOXF2, and SPRY1 and increased mRNA expression of CCL2 and IL-6. N = 6, one-way ANOVA. **(B)** TNF-*α* treatment significantly reduced PDGFR-*α* protein expression.

We have previously reported that mesenchymal stromal cells (MSCs) from tracheal aspirates of very premature infants, mechanically ventilated for respiratory distress syndrome in the first week of life, show surface markers, colony-forming capacity, and differentiation potential consistent with an alveolar mesenchymal cell progenitor capable of differentiation into myofibroblast (48, 63, 64). We have also shown that MSCs from infants developing BPD show lower PDGFR-*α* expression (60). Upon review of the MSC gene expression data we noted that these cells express the TNF-*α* receptor TNF receptor type 1 (TNFR1), suggesting these cells have the capacity to respond to TNF-*α*. TNF-*α* inhibits PDGFR-*α* expression in mouse embryonic fibroblasts (65). Therefore, next we assessed the effect of TNF-*α* on preterm lung MSC expression of PDGFR-*α* and other related genes. MSC lines from six premature infants were incubated with TNF-*α* for 48 h. TNF-*α* decreased the mRNA and protein expression of PDGFRA (**Figures 8A, B**). Also decreased was the mRNA expression of WNT2, FOXF2, and SPRY1 genes (**Figure 8A**) required for normal mesenchymal cell function and lung development (66). TNF-*α* increased the mRNA expression of CCL2 and IL-6 (**Figure 6A**), creating a possible feed forward mechanism to promote inflammation and recruitment of inflammatory cells.

### TNF-*α* Protein Levels in Preterm Infant Airways

**Figure 9:**
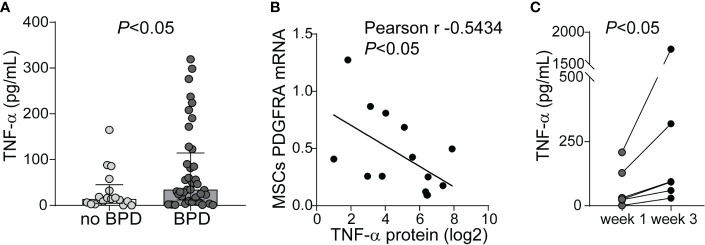
TNF-*α* protein levels in tracheal aspirates from human infants receiving mechanical ventilation for respiratory distress syndrome. **(A)** Tracheal aspirates were collected in the first week of life. Supernatant was assayed for TNF-*α*. Comparison by Mann–Whitney test. **(B)** Tracheal aspirate TNF-*α* protein levels (log2-transformed) positively correlated with MSCs PDGFRA mRNA expression. **(C)** For patients who remained endotracheally intubated receiving mechanical ventilation tracheal aspirates were obtained during week 1 and week 3 of mechanical ventilation, and TNF-*α* levels were quantified. Statistical significance was pointed by Wilcoxon matched-pairs test.

Prior studies demonstrate elevated TNF-*α* levels in the airway fluid of infants with evolving and established BPD (26). We sought to confirm these findings in a larger cohort of premature infants receiving mechanical ventilation for respiratory distress syndrome in the first week of life. We collected tracheal aspirates from 54 premature infants and assayed the supernatants for TNF-*α* protein. Characteristics of the patients are described in **Table 1**. We defined BPD based on need for oxygen supplementation at 36 weeks postmenstrual age. We did not have information if death before 36 weeks postmenstrual age was owing to persistent parenchymal lung disease and respiratory failure that cannot be attributable to other neonatal morbidities. For prematurely born infants, death before 36 weeks postmenstrual age is a competing outcome for BPD and may confound a cause-specific analysis, therefore we combined both outcomes. TNF-*α* protein levels were significantly higher in aspirates from premature infants developing BPD compared to aspirates from infants who did not develop BPD (**Figure 9A**). We also analyzed the relationship between TNF-*α* protein levels in the tracheal aspirate supernatant with PDGFR-*α* mRNA expression of MSCs, isolated from the same tracheal aspirate. We found a significant negative correlation between tracheal aspirate TNF-*α* protein levels and MSC PDGFR-*α* mRNA expression (**Figure 9B**). For infants who continued to receive mechanical ventilation for at least 3 weeks, we found a significant increase in tracheal aspirate TNF-*α* protein levels from week 1 to week 3 of mechanical ventilation (**Figure 9C**).

**Table 1:** Baseline characteristics of infants who developed BPD or died before 36 weeks postmenstrual age (BPD/death), and infants who did not develop BPD (No BPD).

	BPD/death (N = 37)	No BPD (N = 17)	P-value
Male gender, n (% total)	23 (62)	12 (71)	0.76
Gestational Age, weeks, mean ± SD	25.9 ± 1.7	29.4 ± 1.7	<0.0001
Birth weight, grams, mean ± SD	871 ± 301	1361 ± 308	<0.0001
Postnatal Age at Sample Collection, days, median (IQR)	3 (3)	2 (2)	<0.05
MSC+, n (% total)	27 (73)	12 (71)	>0.99
Suspected clinical chorioamnionitis, n (% total)	9 (24)	1 (6)	0.14
Antenatal steroids, n (% total)	26 (70)	9 (53)	0.24
Vent days, median (IQR)	47 (52)	5 (9.5)	<0.0001
O2 days, median (IQR)	216 (245.5)	24 (37.25)	<0.0001

IQR, interquartile range; Vent days, days requiring mechanical ventilations; O2 days, days requiring supplemental oxygen. Gestational age and birth weight were compared using Student’s t-test. Postnatal age at sample collection, vent days and O2 days were compared using Wilcoxon–Mann–Whitney test. Gender, frequency of MSCs, suspected clinical chorioamnionitis, antenatal steroids were compared using Fisher’s exact test.

## Discussion

In the current study, we used an animal model with repeated administration of LPS into the airways of immature mice to simulate prolonged airway exposure to gram-negative bacteria and assess the effects on pulmonary alveolarization, as well as inflammation, focusing on lung cytokine expression and inflammatory cell trafficking. Repetitive LPS exposure of immature mice induced persistent hypoalveolarization lasting up to 18 days after the last LPS administration. Also, LPS upregulated the expression of lung pro-inflammatory cytokines (TNF-*α*, IL-17a, IL-6, IL-1*β*) and chemokines (CCL2, CCL7, CXCL1, and CXCL2), while the expression of genes involved in lung alveolar and mesenchymal cell development (PDGFR-*α*, FGF7, FGF10, and SPRY1) was decreased. In addition, LPS induced recruitment of ExMf, including CCR2+ ExMf, as well as other myeloid cells like DCs and neutrophils. CCR2−/− mice were protected from LPS-induced hypoalveolarization, increased interstitial *α*-SMA expression, and reduced PDGFR*α* expression at the tips of the secondary alveolar crests. The number of ExMf recruited to the lungs in response to repetitive LPS exposure was significantly lower in CCR2−/− mice, compared to wild type mice. Also, unlike wild type mice, the number of TNF-*α*-producing ExMf, as well as whole lung TNF-*α* mRNA expression after LPS remained low and unchanged in CCR2−/− mice. Further, in wild type mice, pharmacological inhibition of TLR4 with TAK-242 blocked the effect of LPS on alveolarization, *α*-SMA and PDGFR*α* expression. Finally, in an *in vitro* study, TNF-*α* and IL-17a induced *α*-smooth muscle actin expression in the distal airspaces of E16 fetal mouse lung explants. TNF-*α* decreased human preterm lung MSC mRNA and protein expression of PDGFR-*α* and reduced mRNA expression of WNT2, FOXF2, and SPRY1. Collectively, our findings demonstrate that in immature mice repetitive LPS exposure, through TLR4 signaling increases lung inflammation and impairs lung alveolar growth in a CCR2-dependent manner.

Inflammation typically precedes the clinical symptoms of BPD (67). However, the specific immunologic mechanisms leading to impaired alveolar growth, the hallmark of BPD histopathology are unknown. Premature infants who develop BPD have higher tracheal aspirate chemokine levels of CCL2, CCL7, and CCL8, suggesting a role for these mediators in the pathogenesis of BPD (21, 68). CCL2, CCL7, and CCL8 are ligands for CCR2, a chemokine receptor that mediates recruitment of inflammatory cells, among which inflammatory monocytes, exudative macrophages and other innate immune cells (38, 69, 70). CCR2 signaling has been implicated in other lung diseases with interstitial involvement, including pulmonary fibrosis (38) and pediatric interstitial lung disease (71). Animal studies implicate a role for CCL2–CCR2 signaling in alveolar development. In murine models of BPD, both hyperoxia and LPS exposure of immature mice induce higher lung levels of CCL2 (13, 27). CCL2 is required for hyperoxia-induced alveolar simplification (27). Further, hyperoxia-induced lung injury and hypoalveolarization and exudative macrophage recruitment are attenuated in CCR2−/− mice (40, 72). The inflammatory stimulus during hyperoxic exposure is likely multifaceted. The development of a model of postnatal LPS exposure-induced hypoalveolarization (13, 42) confirms a relationship between the bacteria-derived inflammatory stimulus and impaired alveolar development. Our model offers the opportunity to investigate the mechanisms by which inflammation induced by repeated LPS administration into the airway translates into hypoalveolarization. Specifically, our findings that LPS-induced expression of lung CCL2 and CCL7 chemokines precede the development of hypoalveolarization are reminiscent of patterns of chemokine expression observed in human infants developing BPD (21, 67, 68). To our knowledge, in this study, we demonstrate for the first time the requirement of CCR2 for repeated LPS exposure-induced hypoalveolarization, changes in interstitial *α*-SMA and PDGFR*α* expression, as well as pulmonary inflammation and exudative macrophage accumulation in immature mice.

Inflammatory stimuli induce CCL2 and CCR2 expression in multiple cell types, including macrophages, monocytes, as well as epithelial, endothelial, smooth muscle cells, and fibroblasts (73–75). We evaluated the cellular inflammatory response induced by LPS focusing on ExMf, myeloid cells recruited in CCR2-dependent manner (34, 37). The LPS-induced increase in ExMf and CCR2+ ExMf correlated with an increase in CCL2 mRNA and protein and CCL7 mRNA, both transcriptionally regulated ligands for CCR2. The higher magnitude of increase in ExMf and CCR2+ ExMf after repetitive LPS stimulation present at the same time when hypoalveolarization is observed further affirms our interest in the hypothesis that recruited inflammatory cells, *i.e.* macrophages contribute to impaired alveolar development (27). Compared to wild type mice, the number of ExMf recruited to the lungs in response to repetitive LPS exposure was significantly lower in CCR2−/− mice. However, the ExMf response to LPS in CCR2−/− mice was not completely blocked. This finding is in agreement with other reports demonstrating attenuated, but not completely abolished recruitment of exudative macrophages in a murine model of hyperoxia-induced hypoalveolarization (40, 72), and non-infectious lung injury model (37). In the hyperoxia-induced hypoalveolarization model, other resident macrophage populations that become activated upon exposure, specifically Csf1r-expressing monocyte/macrophages, but not neutrophils are key mediators of arrested alveolarization (40). It is possible that other chemokines or chemokine receptors are responsible for the recruitment of the remaining exudative macrophages. For example, CCR5 is also required for recruitment of ExMf into inflamed issues (76). CCR2 and CCR5 have opposing effects in response to influenza A infection, and while CCR2 deficiency dampens the inflammatory response, CCR5 deficiency leads to a hyperacute inflammatory response (77).

Our findings that pharmacological inhibition of TLR4 with TAK-242 blocked the effects of LPS on alveolarization, *α*-SMA and PDGFR*α* expression highlight a potential therapeutic implication for TLR4 inhibitors to preserve alveolarization and attenuate interstitial fibrosis in the immature lung exposed to gram-negative bacterial inflammation. TAK-242 prevents bleomycin-induced dermal and pulmonary fibrosis and mitigates increased collagen and *α*-SMA expression in cultured fibroblasts stimulated with fibronectin extra domain A isoform, a profibrotic treatment to simulate systemic sclerosis (78). The specific mechanisms by which TLR4-dependent signals regulate PDGFR*α* expression, promote abnormal myofibroblast differentiation, and impair lung alveolar growth are incompletely understood and will be the focus of our future studies.

When administered systemically or into the airways LPS is a potent stimulator of pro-inflammatory cytokines, including TNF-*α*, IL-6, IL-1*β* (42, 79). Our findings of higher lung levels of TNF-*α*, IL-6 and IL-1*β* following LPS administration into the airways are reminiscent of the higher levels of these cytokines in tracheal aspirates from premature infants with evolving BPD (19, 26). Further evidence implicating TNF-*α* in BPD development is provided by a study, which demonstrates that the TNF-*α* −238 allele, associated with lower production of TNF-*α* upon stimulation, reduces the risk and severity of BPD (80). *In vitro* studies demonstrate marked inhibitory effects of TNF-*α* on the expression of pulmonary surfactant proteins, SP-A and SP-B (81), and thus support the concept that TNF-*α* may contribute in the pathogenesis of respiratory distress syndrome, which often precedes BPD development. Furthermore, in mouse embryonic fibroblasts, TNF-*α* reduces the expression of PDGFR*α*, a receptor absolutely required for lung alveolar development (49, 65). In this context, our findings in fetal mouse lung explants, human preterm lung MSCs and preterm infants’ tracheal aspirates further extend the implication of TNF-*α* in negative control of PDGFR*α* expression, abnormal myofibroblast differentiation in the distal airways and impaired lung alveolar growth. However, we note that our findings are in contrast to a study reporting that the absence of TNF-*α* enhances the inflammatory response in the newborn lung undergoing mechanical ventilation with oxygen-rich gas (82). In addition, unlike our and other studies (26) showing higher levels of TNF-*α* in tracheal aspirates from infants with evolving BPD, in this study tracheal aspirate TNF-*α* levels were lower in infants with moderate to severe BPD (82). Several factors could account for the observed differences. In regard to the animal studies these include differences in the models, timing when the responses were analyzed, balance between absent or enhanced inflammatory response. For example, a certain amount of inflammation is required for resolution of the response *versus* detrimental effects from sustained or continuous inflammatory stimulus. The contradictory human tracheal aspirate TNF-*α* results could be due to differences when the tracheal aspirates were collected, at birth in the study by Ehrhardt et al. (82) *versus* the first week of life in our study.

We also acknowledge that focusing on a single cytokine, *i.e.* TNF-*α* is a limitation of our study and may underscore the importance of other secreted or cell-bound signals, other innate or adaptive immune responses, cell–cell or cell–matrix interactions. Another cytokine that was increased by LPS in immature lungs was IL-17a. IL-17a, a member of the IL-17 family, is a pro-inflammatory cytokine produced by T cells (T helper 17 cells), *γδ* T cells, as well as innate lymphocytes, and myeloid cells, such as neutrophils and macrophages (83–85). Interestingly, histological chorioamnionitis, a risk factor for BPD development, is associated with a higher frequency of IL-17a-expressing T lymphocytes in cord blood (86). The main source of IL-17a in mucosal tissues, including lungs, is *γδ* T cells (87), which can also be recruited in a CCR2-dependent manner (88). While in our study IL-17a did not have a direct effect on MSC expression, in the murine fetal lung explants IL-17a, as well as TNF-*α* increased *α*-smooth muscle actin expression. We attribute this to an indirect effect through an intermediate cell in the explant model. Our future studies will focus on the role of IL-17a-producing innate immune cells, including *γδ* T cells in LPS-induced hypoalveolarization. In addition, other pro-inflammatory cytokines, including IL-1*β* and IL-6 may also have an effect on MSCs. Future studies are needed to investigate the effects of these and other pro-inflammatory cytokines on lung development and mesenchymal cell function.

In conclusion, our results demonstrate that in immature mice, repetitive LPS exposure, through TLR4 signaling increases lung inflammation and impairs lung alveolar growth in a CCR2-dependent manner. This mechanism links gram-negative bacterial stimulation of the innate immune system to alveolar development. This helps to further understand the mechanisms of BPD development and chronic airway disease in survivors of preterm birth.

## Data Availability Statement

All datasets presented in this study are included in the article/**Supplementary Material**.

## Ethics Statement

The studies involving human participants were reviewed and approved by the University of Michigan Medical School Institutional Review Board. Written informed consent to participate in this study was provided by the participants’ legal guardian/next of kin. The animal study was reviewed and approved by the University of Michigan Institutional Animal Care and Use Committee.

## Author Contributions

TC and AP conceived and designed the experiments, analyzed the data, and wrote the paper. TC, AB, CF, Y-JZ, and LR performed the experiments, collected samples, interpreted data, edited and reviewed the manuscript. AG collected samples, performed experiments, and interpreted data. BM interpreted data and reviewed the manuscript. All authors contributed to the article and approved the submitted version.

## Funding

This work was supported by NIH grant R01 HL140572 and the Amendt-Heller Award for Newborn Research, Department of Pediatrics, University of Michigan.

## Conflict of Interest

The authors declare that the research was conducted in the absence of any commercial or financial relationships that could be construed as a potential conflict of interest.

## References

[1] Stoll BJ Hansen NI Bell EF Walsh MC Carlo WA Shankaran S Trends in Care Practices, Morbidity, and Mortality of Extremely Preterm Neonates, 1993-2012. Jama (2015) 314(10):1039–51. 10.1001/jama.2015.10244 PMC4787615 26348753

[2] Cotten CM Oh W McDonald S Carlo W Fanaroff AA Duara S Prolonged hospital stay for extremely premature infants: risk factors, center differences, and the impact of mortality on selecting a best-performing center. J Perinatol (2005) 25(10):650–5. 10.1038/sj.jp.7211369 16079906

[3] Been JV Lugtenberg MJ Smets E van Schayck CP Kramer BW Mommers M Preterm birth and childhood wheezing disorders: a systematic review and meta-analysis. PloS Med (2014) 11(1):e1001596. 10.1371/journal.pmed.1001596 24492409 PMC3904844

[4] Toti P Buonocore G Tanganelli P Catella AM Palmeri ML Vatti R Bronchopulmonary dysplasia of the premature baby: an immunohistochemical study. Pediatr Pulmonol (1997) 24:22–8. 10.1002/(SICI)1099-0496(199707)24:1<22::AID-PPUL4>3.0.CO;2-L 9261849

[5] Kallapur SG Jobe AH Contribution of inflammation to lung injury and development. Arch Dis Child Fetal Neonatal Ed (2006) 91(2):F132–5. 10.1136/adc.2004.068544 PMC2672671 16492951

[6] Wright CJ Kirpalani H Targeting inflammation to prevent bronchopulmonary dysplasia: can new insights be translated into therapies? Pediatrics (2011) 128(1):111–26. 10.1542/peds.2010-3875 PMC3124103 21646264

[7] Hartling L Liang Y Lacaze-Masmonteil T Chorioamnionitis as a risk factor for bronchopulmonary dysplasia: a systematic review and meta-analysis. Arch Dis Child Fetal Neonatal Ed (2012) 97(1):F8–f17. 10.1136/adc.2010.210187 21697236

[8] Ballard AR Mallett LH Pruszynski JE Cantey JB Chorioamnionitis and subsequent bronchopulmonary dysplasia in very-low-birth weight infants: a 25-year cohort. J Perinatol (2016) 36(12):1045–8. 10.1038/jp.2016.138 27583395

[9] Lapcharoensap W Gage SC Kan P Profit J Shaw GM Gould JB Hospital variation and risk factors for bronchopulmonary dysplasia in a population-based cohort. JAMA Pediatr (2015) 169(2):e143676. 10.1001/jamapediatrics.2014.3676 25642906

[10] Viscardi RM Muhumuza CK Rodriguez A Fairchild KD Sun CC Gross GW Inflammatory markers in intrauterine and fetal blood and cerebrospinal fluid compartments are associated with adverse pulmonary and neurologic outcomes in preterm infants. Pediatr Res (2004) 55(6):1009–17. 10.1203/01.pdr.0000127015.60185.8a 15155869

[11] Yoon BH Romero R Jun JK Park KH Park JD Ghezzi F Amniotic fluid cytokines (interleukin-6, tumor necrosis factor-alpha, interleukin-1 beta, and interleukin-8) and the risk for the development of bronchopulmonary dysplasia. Am J Obstetrics Gynecol (1997) 177(4):825–30. 10.1016/S0002-9378(97)70276-X 9369827

[12] Beeton ML Maxwell NC Davies PL Nuttall D McGreal E Chakraborty M Role of pulmonary infection in the development of chronic lung disease of prematurity. Eur Respir J (2011) 37(6):1424–30. 10.1183/09031936.00037810 20884745

[13] Lal CV Olave N Travers C Rezonzew G Dolma K Simpson A Exosomal microRNA predicts and protects against severe bronchopulmonary dysplasia in extremely premature infants. JCI Insight (2018) 3(5):e93994. 10.1172/jci.insight.93994 PMC5922295 29515035

[14] Mourani PM Harris JK Sontag MK Robertson CE Abman SH Molecular identification of bacteria in tracheal aspirate fluid from mechanically ventilated preterm infants. PloS One (2011) 6(10):e25959. 10.1371/journal.pone.0025959 22016793 PMC3189942

[15] Cordero L Ayers LW Davis K Neonatal airway colonization with gram-negative bacilli: association with severity of bronchopulmonary dysplasia. Pediatr Infect Dis J (1997) 16(1):18–23. 10.1097/00006454-199701000-00005 9002095

[16] Tramper J Zhang H Foglia EE Dysart KC Padula MA Sullivan KV The Association between Positive Tracheal Aspirate Cultures and Adverse Pulmonary Outcomes in Preterm Infants with Severe Bronchopulmonary Dysplasia. Am J Perinatol (2017) 34(1):96–104. 10.1055/s-0036-1584541 27285471

[17] Merritt TA Stuard ID Puccia J Wood B Edwards DK Finkelstein J Newborn tracheal aspirate cytology: classification during respiratory distress syndrome and bronchopulmonary dysplasia. J Pediatr (1981) 98(6):949–56. 10.1016/S0022-3476(81)80603-8 7229802

[18] Merritt TA Puccia JM Stuard ID Cytologic evaluation of pulmonary effluent in neonates with respiratory distress syndrome and bronchopulmonary dysplasia. Acta Cytol (1981) 25(6):631–9. 6947668

[19] Eldredge LC Creasy RS Presnell S Debley JS Juul SE Mayock DE Infants with evolving bronchopulmonary dysplasia demonstrate monocyte-specific expression of IL-1 in tracheal aspirates. Am J Physiol Lung Cell Mol Physiol (2019) 317(1):L49–l56. 10.1152/ajplung.00060.2019 30969811

[20] Clement A Chadelat K Sardet A Grimfeld A Tournier G Alveolar macrophage status in bronchopulmonary dysplasia. Pediatr Res (1988) 23(5):470–3. 10.1203/00006450-198805000-00007 3387169

[21] Baier RJ Majid A Parupia H Loggins J Kruger TE CC chemokine concentrations increase in respiratory distress syndrome and correlate with development of bronchopulmonary dysplasia. Pediatr Pulmonol (2004) 37(2):137–48. 10.1002/ppul.10417 14730659

[22] Ozdemir A Brown MA Morgan WJ Markers and mediators of inflammation in neonatal lung disease. Pediatr Pulmonol (1997) 23(4):292–306. 10.1002/(SICI)1099-0496(199704)23:4<292::AID-PPUL7>3.0.CO;2-O 9141115

[23] Kotecha S Wilson L Wangoo A Silverman M Shaw RJ Increase in interleukin (IL)-1 beta and IL-6 in bronchoalveolar lavage fluid obtained from infants with chronic lung disease of prematurity. Pediatr Res (1996) 40(2):250–6. 10.1203/00006450-199608000-00010 8827773

[24] Kotecha S Chan B Azam N Silverman M Shaw RJ Increase in interleukin-8 and soluble intercellular adhesion molecule-1 in bronchoalveolar lavage fluid from premature infants who develop chronic lung disease. Arch Dis Child Fetal Neonatal Ed (1995) 72(2):F90–6. 10.1136/fn.72.2.F90 PMC2528395 7712280

[25] Ambalavanan N Carlo WA D’Angio CT McDonald SA Das A Schendel D Cytokines associated with bronchopulmonary dysplasia or death in extremely low birth weight infants. Pediatrics (2009) 123(4):1132–41. 10.1542/peds.2008-0526 PMC2903210 19336372

[26] Jonsson B Tullus K Brauner A Lu Y Noack G Early increase of TNF alpha and IL-6 in tracheobronchial aspirate fluid indicator of subsequent chronic lung disease in preterm infants. Arch Dis Child Fetal Neonatal Ed (1997) 77(3):F198–201. 10.1136/fn.77.3.F198 PMC1720706 9462189

[27] Anyanwu AC Bentley JK Popova AP Malas O Alghanem H Goldsmith AM Suppression of inflammatory cell trafficking and alveolar simplification by the heme oxygenase-1 product carbon monoxide. Am J Physiol Lung Cell Mol Physiol (2014) 306(8):L749–63. 10.1152/ajplung.00236.2013 PMC3989725 24532288

[28] Nold MF Mangan NE Rudloff I Cho SX Shariatian N Samarasinghe TD Interleukin-1 receptor antagonist prevents murine bronchopulmonary dysplasia induced by perinatal inflammation and hyperoxia. Proc Natl Acad Sci U S A (2013) 110(35):14384–9. 10.1073/pnas.1306859110 PMC3761642 23946428

[29] Yoder BA Siler-Khodr T Winter VT Coalson JJ High-frequency oscillatory ventilation: effects on lung function, mechanics, and airway cytokines in the immature baboon model for neonatal chronic lung disease. Am J Respir Crit Care Med (2000) 162(5):1867–76. 10.1164/ajrccm.162.5.9912145 11069828

[30] Heine H Rietschel ET Ulmer AJ The biology of endotoxin. Mol Biotechnol (2001) 19(3):279–96. 10.1385/MB:19:3:279 11721624

[31] Park BS Song DH Kim HM Choi BS Lee H Lee JO The structural basis of lipopolysaccharide recognition by the TLR4-MD-2 complex. Nature (2009) 458(7242):1191–5. 10.1038/nature07830 19252480

[32] Shi J Zhao Y Wang Y Gao W Ding J Li P Inflammatory caspases are innate immune receptors for intracellular LPS. Nature (2014) 514(7521):187–92. 10.1038/nature13683 25119034

[33] Juskewitch JE Knudsen BE Platt JL Nath KA Knutson KL Brunn GJ LPS-induced murine systemic inflammation is driven by parenchymal cell activation and exclusively predicted by early MCP-1 plasma levels. Am J Pathol (2012) 180(1):32–40. 10.1016/j.ajpath.2011.10.001 22067909 PMC3338351

[34] Maus UA Wellmann S Hampl C Kuziel WA Srivastava M Mack M CCR2-positive monocytes recruited to inflamed lungs downregulate local CCL2 chemokine levels. Am J Physiol Lung Cell Mol Physiol (2005) 288(2):L350–8. 10.1152/ajplung.00061.2004 15516494

[35] Tsou CL Peters W Si Y Slaymaker S Aslanian AM Weisberg SP Critical roles for CCR2 and MCP-3 in monocyte mobilization from bone marrow and recruitment to inflammatory sites. J Clin Invest (2007) 117(4):902–9. 10.1172/JCI29919 PMC1810572 17364026

[36] Gurczynski SJ Nathani N Warheit-Niemi HI Hult EM Podsiad A Deng J CCR2 mediates increased susceptibility to post-H1N1 bacterial pneumonia by limiting dendritic cell induction of IL-17. Mucosal Immunol (2019) 12(2):518–30. 10.1038/s41385-018-0106-4 PMC6375750 30498200

[37] Tighe RM Liang J Liu N Jung Y Jiang D Gunn MD Recruited exudative macrophages selectively produce CXCL10 after noninfectious lung injury. Am J Respir Cell Mol Biol (2011) 45(4):781–8. 10.1165/rcmb.2010-0471OC PMC3208617 21330464

[38] Moore BB Paine R,3 Christensen PJ Moore TA Sitterding S Ngan R Protection from pulmonary fibrosis in the absence of CCR2 signaling. J Immunol (2001) 167(8):4368–77. 10.4049/jimmunol.167.8.4368 11591761

[39] Osterholzer JJ Olszewski MA Murdock BJ Chen GH Erb-Downward JR Subbotina N Implicating exudate macrophages and Ly-6C(high) monocytes in CCR2-dependent lung fibrosis following gene-targeted alveolar injury. J Immunol (2013) 190(7):3447–57. 10.4049/jimmunol.1200604 PMC3608799 23467934

[40] Kalymbetova TV Selvakumar B Rodriguez-Castillo JA Gunjak M Malainou C Heindl MR Resident alveolar macrophages are master regulators of arrested alveolarization in experimental bronchopulmonary dysplasia. J Pathol (2018) 245(2):153–9. 10.1002/path.5076 29574785

[41] You Y Guo C Zhang H Deng S Tang J Xu L Effect of Intranasal Instillation of Lipopolysaccharide on Lung Development and Its Related Mechanism in Newborn Mice. J Interferon Cytokine Res (2019) 39(11):684–93. 10.1089/jir.2019.0006 PMC6820870 31268385

[42] Shrestha AK Bettini ML Menon RT Gopal VYN Huang S Edwards DP Consequences of early postnatal lipopolysaccharide exposure on developing lungs in mice. Am J Physiol Lung Cell Mol Physiol (2019) 316(1):L229–l44. 10.1152/ajplung.00560.2017 PMC6383495 30307313

[43] Nguyen L Castro O De Dios R Sandoval J McKenna S Wright CJ Sex-differences in LPS-induced neonatal lung injury. Sci Rep (2019) 9(1):8514. 10.1038/s41598-019-44955-0 31186497 PMC6560218

[44] Nakano H Free ME Whitehead GS Maruoka S Wilson RH Nakano K Pulmonary CD103(+) dendritic cells prime Th2 responses to inhaled allergens. Mucosal Immunol (2012) 5(1):53–65. 10.1038/mi.2011.47 22012243 PMC3697034

[45] Scherle W A simple method for volumetry of organs in quantitative stereology. Mikroskopie (1970) 26(1):57–60. 5530651

[46] Schneider JP Ochs M Stereology of the lung. Methods Cell Biol (2013) 113:257–94. 10.1016/B978-0-12-407239-8.00012-4 23317906

[47] Hsia CC Hyde DM Ochs M Weibel ER An official research policy statement of the American Thoracic Society/European Respiratory Society: standards for quantitative assessment of lung structure. Am J Respir Crit Care Med (2010) 181(4):394–418. 10.1164/rccm.200809-1522ST 20130146 PMC5455840

[48] Hennrick KT Keeton AG Nanua S Kijek TG Goldsmith AM Sajjan US Lung cells from neonates show a mesenchymal stem cell phenotype. Am J Respir Crit Care Med (2007) 175:1158–64. 10.1164/rccm.200607-941OC 17332484

[49] Boström H Willetts K Pekny M Levéen P Lindahl P Hedstrand H PDGF-A Signaling Is a Critical Event in Lung Alveolar Myofibroblast Development and Alveogenesis. Cell (1996) 85(6):863–73. 10.1016/S0092-8674(00)81270-2 8681381

[50] Boström H Gritli-Linde A Betsholtz C PDGF-a/PDGF alpha-receptor signaling is required for lung growth and the formation of alveoli but not for early lung branching morphogenesis. Dev Dynamics (2002) 223(1):155–62. 10.1002/dvdy.1225 11803579

[51] Yamamoto S Fukumoto E Yoshizaki K Iwamoto T Yamada A Tanaka K Platelet-derived growth factor receptor regulates salivary gland morphogenesis via fibroblast growth factor expression. J Biol Chem (2008) 283(34):23139–49. 10.1074/jbc.M710308200 18559345

[52] Padela S Yi M Cabacungan J Shek S Belcastro R Masood A A critical role for fibroblast growth factor-7 during early alveolar formation in the neonatal rat. Pediatr Res (2008) 63(3):232–8. 10.1203/PDR.0b013e31815f6e3a 18091341

[53] Toriseva M Ala-aho R Peltonen S Peltonen J Grenman R Kahari VM Keratinocyte growth factor induces gene expression signature associated with suppression of malignant phenotype of cutaneous squamous carcinoma cells. PloS One (2012) 7(3):e33041. 10.1371/journal.pone.0033041 22427941 PMC3299721

[54] Hashimoto S Nakano H Singh G Katyal S Expression of Spred and Sprouty in developing rat lung. Mech Dev (2002) 119(Suppl 1):S303–9. 10.1016/S0925-4773(03)00132-1 14516701

[55] Bedoret D Wallemacq H Marichal T Desmet C Quesada Calvo F Henry E Lung interstitial macrophages alter dendritic cell functions to prevent airway allergy in mice. J Clin Invest (2009) 119(12):3723–38. 10.1172/JCI39717 PMC2786798 19907079

[56] Lindahl P Karlsson L Hellstrom M Gebre-Medhin S Willetts K Heath J Alveogenesis failure in PDGF-A-deficient mice is coupled to lack of distal spreading of alveolar smooth muscle cell progenitors during lung development. Development (1997) 124(20):3943–53. 10.1242/dev.124.20.3943 9374392

[57] Noguchi A Reddy R Kursar JD Parks WC Mecham RP Smooth muscle isoactin and elastin in fetal bovine lung. Exp Lung Res (1989) 15:537–52. 10.3109/01902148909069617 2767003

[58] Yamada M Kurihara H Kinoshita K Sakai T Temporal Expression of Alpha-Smooth Muscle Actin and Drebrin in Septal Interstitial Cells during Alveolar Maturation. J Histochem Cytochem (2005) 53(6):735–44. 10.1369/jhc.4A6483.2005 15928322

[59] Hirakawa H Pierce RA Bingol-Karakoc G Karaaslan C Weng M Shi G-P Cathepsin S Deficiency Confers Protection from Neonatal Hyperoxia-induced Lung Injury. Am J Respir Crit Care Med (2007) 176(8):778–85. 10.1164/rccm.200704-519OC PMC2020827 17673697

[60] Popova AP Bentley JK Cui TX Richardson MN Linn MJ Lei J Reduced platelet-derived growth factor receptor expression is a primary feature of human bronchopulmonary dysplasia. Am J Physiol Lung Cell Mol Physiol (2014) 307(3):L231–9. 10.1152/ajplung.00342.2013 PMC4121643 24907056

[61] Matsunaga N Tsuchimori N Matsumoto T Ii M TAK-242 (resatorvid), a small-molecule inhibitor of Toll-like receptor (TLR) 4 signaling, binds selectively to TLR4 and interferes with interactions between TLR4 and its adaptor molecules. Mol Pharmacol (2011) 79(1):34–41. 10.1124/mol.110.068064 20881006

[62] Benjamin JT Smith RJ Halloran BA Day TJ Kelly DR Prince LS FGF-10 is decreased in bronchopulmonary dysplasia and suppressed by Toll-like receptor activation. Am J Physiol Lung Cell Mol Physiol (2007) 292(2):L550–8. 10.1152/ajplung.00329.2006 17071719

[63] Popova AP Bozyk PD Goldsmith AM Linn MJ Lei J Bentley JK Autocrine production of TGF-beta1 promotes myofibroblastic differentiation of neonatal lung mesenchymal stem cells. Am J Physiol Lung Cell Mol Physiol (2010) 298(6):L735–43. 10.1152/ajplung.00347.2009 PMC2886615 20190033

[64] Bozyk PD Popova AP Bentley JK Goldsmith AM Linn MJ Weiss DJ Mesenchymal stromal cells from neonatal tracheal aspirates demonstrate a pattern of lung-specific gene expression. Stem Cells Dev (2011) 20(11):1995–2007. 10.1089/scd.2010.0494 21341990 PMC3202893

[65] Zhang N Chan CW Sanchez-Guerrero E Khachigian LM Repression of PDGF-R-alpha after cellular injury involves TNF-alpha, formation of a c-Fos-YY1 complex, and negative regulation by HDAC. Am J Physiol Cell Physiol (2012) 302(11):C1590–8. 10.1152/ajpcell.00429.2011 22322974

[66] Fulton CT Cui TX Goldsmith AM Bermick J Popova AP Gene Expression Signatures Point to a Male Sex-Specific Lung Mesenchymal Cell PDGF Receptor Signaling Defect in Infants Developing Bronchopulmonary Dysplasia. Sci Rep (2018) 8(1):17070. 10.1038/s41598-018-35256-z 30459472 PMC6244280

[67] Leroy S Caumette E Waddington C Hebert A Brant R Lavoie PM A Time-Based Analysis of Inflammation in Infants at Risk of Bronchopulmonary Dysplasia. J Pediatr (2018) 192:60–5.e1. 10.1016/j.jpeds.2017.09.011 29092751

[68] Baier RJ Loggins J Kruger TE Monocyte chemoattractant protein-1 and interleukin-8 are increased in bronchopulmonary dysplasia: relation to isolation of Ureaplasma urealyticum. J Invest Med Off Publ Am Fed Clin Res (2001) 49(4):362–9. 10.2310/6650.2001.33902 11478413

[69] Jia T Serbina NV Brandl K Zhong MX Leiner IM Charo IF Additive roles for MCP-1 and MCP-3 in CCR2-mediated recruitment of inflammatory monocytes during Listeria monocytogenes infection. J Immunol (2008) 180(10):6846–53. 10.4049/jimmunol.180.10.6846 PMC2386263 18453605

[70] Fujimura N Xu B Dalman J Deng H Aoyama K Dalman RL CCR2 inhibition sequesters multiple subsets of leukocytes in the bone marrow. Sci Rep (2015) 5:11664. 10.1038/srep11664 26206182 PMC4513281

[71] Hartl D Griese M Nicolai T Zissel G Prell C Reinhardt D A role for MCP-1/CCR2 in interstitial lung disease in children. Respir Res (2005) 6:93. 10.1186/1465-9921-6-93 16095529 PMC1199626

[72] Eldredge LC Creasy RS Tanaka S Lai JF Ziegler SF Imbalance of Ly-6C(hi) and Ly-6C(lo) Monocytes/Macrophages Worsens Hyperoxia-Induced Lung Injury and Is Rescued by IFN-gamma. J Immunol (2019) 202(9):2772–81. 10.4049/jimmunol.1801374 30944158

[73] Hildebrandt GC Duffner UA Olkiewicz KM Corrion LA Willmarth NE Williams DL A critical role for CCR2/MCP-1 interactions in the development of idiopathic pneumonia syndrome after allogeneic bone marrow transplantation. Blood (2004) 103(6):2417–26. 10.1182/blood-2003-08-2708 14615370

[74] Girkin J Hatchwell L Foster P Johnston SL Bartlett N Collison A CCL7 and IRF-7 Mediate Hallmark Inflammatory and IFN Responses following Rhinovirus 1B Infection. J Immunol (2015) 194(10):4924–30. 10.4049/jimmunol.1401362 PMC4417644 25847975

[75] Mercer PF Williams AE Scotton CJ Jose RJ Sulikowski M Moffatt JD Proteinase-activated receptor-1, CCL2, and CCL7 regulate acute neutrophilic lung inflammation. Am J Respir Cell Mol Biol (2014) 50(1):144–57. 10.1165/rcmb.2013-0142OC PMC3930934 23972264

[76] Aggarwal NR King LS D’Alessio FR Diverse macrophage populations mediate acute lung inflammation and resolution. Am J Physiol Lung Cell Mol Physiol (2014) 306(8):L709–25. 10.1152/ajplung.00341.2013 PMC3989724 24508730

[77] Dawson TC Beck MA Kuziel WA Henderson F Maeda N Contrasting effects of CCR5 and CCR2 deficiency in the pulmonary inflammatory response to influenza A virus. Am J Pathol (2000) 156(6):1951–9. 10.1016/S0002-9440(10)65068-7 PMC1850091 10854218

[78] Bhattacharyya S Wang W Tamaki Z Shi B Yeldandi A Tsukimi Y Pharmacological Inhibition of Toll-Like Receptor-4 Signaling by TAK242 Prevents and Induces Regression of Experimental Organ Fibrosis. Front Immunol (2018) 9:2434. 10.3389/fimmu.2018.02434 30405628 PMC6207051

[79] Rittirsch D Flierl MA Day DE Nadeau BA McGuire SR Hoesel LM Acute lung injury induced by lipopolysaccharide is independent of complement activation. J Immunol (2008) 180(11):7664–72. 10.4049/jimmunol.180.11.7664 PMC2753408 18490769

[80] Kazzi SN Kim UO Quasney MW Buhimschi I Polymorphism of tumor necrosis factor-alpha and risk and severity of bronchopulmonary dysplasia among very low birth weight infants. Pediatrics (2004) 114(2):e243–8. 10.1542/peds.114.2.e243 15286263

[81] Wispé JR Clark JC Warner BB Fajardo D Hull WE Holtzman RB Tumor necrosis factor-alpha inhibits expression of pulmonary surfactant protein. J Clin Invest (1990) 86(6):1954–60. 10.1172/JCI114929 PMC329831 2123888

[82] Ehrhardt H Pritzke T Oak P Kossert M Biebach L Förster K Absence of TNF-α enhances inflammatory response in the newborn lung undergoing mechanical ventilation. Am J Physiol Lung Cell Mol Physiol (2016) 310(10):L909–18. 10.1152/ajplung.00367.2015 PMC4896101 27016588

[83] Li L Huang L Vergis AL Ye H Bajwa A Narayan V IL-17 produced by neutrophils regulates IFN-gamma-mediated neutrophil migration in mouse kidney ischemia-reperfusion injury. J Clin Invest (2010) 120(1):331–42. 10.1172/JCI38702 PMC2798679 20038794

[84] Sutton CE Lalor SJ Sweeney CM Brereton CF Lavelle EC Mills KH Interleukin-1 and IL-23 induce innate IL-17 production from gammadelta T cells, amplifying Th17 responses and autoimmunity. Immunity (2009) 31(2):331–41. 10.1016/j.immuni.2009.08.001 19682929

[85] Bosmann M Sarma JV Atefi G Zetoune FS Ward PA Evidence for anti-inflammatory effects of C5a on the innate IL-17A/IL-23 axis. FASEB J (2012) 26(4):1640–51. 10.1096/fj.11-199216 PMC3316904 22202675

[86] Rito DC Viehl LT Buchanan PM Haridas S Koenig JM Augmented Th17-type immune responses in preterm neonates exposed to histologic chorioamnionitis. Pediatr Res (2017) 81(4):639–45. 10.1038/pr.2016.254 PMC5395318 27870827

[87] Lochner M Peduto L Cherrier M Sawa S Langa F Varona R In vivo equilibrium of proinflammatory IL-17+ and regulatory IL-10+ Foxp3+ RORgamma t+ T cells. J Exp Med (2008) 205(6):1381–93. 10.1084/jem.20080034 PMC2413035 18504307

[88] McKenzie DR Kara EE Bastow CR Tyllis TS Fenix KA Gregor CE IL-17-producing gammadelta T cells switch migratory patterns between resting and activated states. Nat Commun (2017) 8:15632. 10.1038/ncomms15632 28580944 PMC5465362

