# Supplementary figures and images for: CCR2 Mediates Chronic LPS-Induced Pulmonary Inflammation and Hypoalveolarization in a Murine Model of Bronchopulmonary Dysplasia
Source: Front Immunol. 2020 Oct 6;11:579628. doi: 10.3389/fimmu.2020.579628 (PMC7573800; doi:10.3389/fimmu.2020.579628)

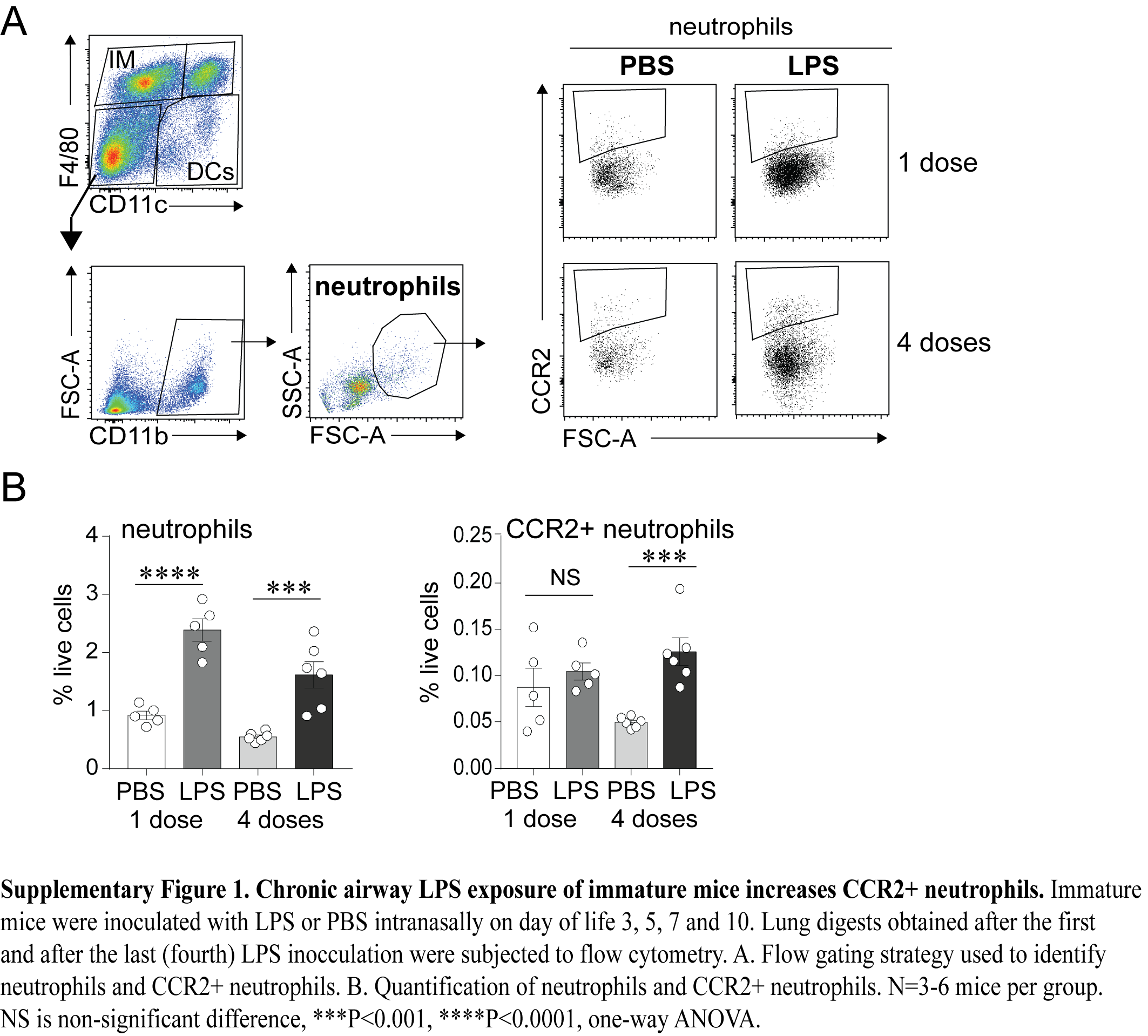

Supplement: Supplementary file 1 [file Image_1.tif]

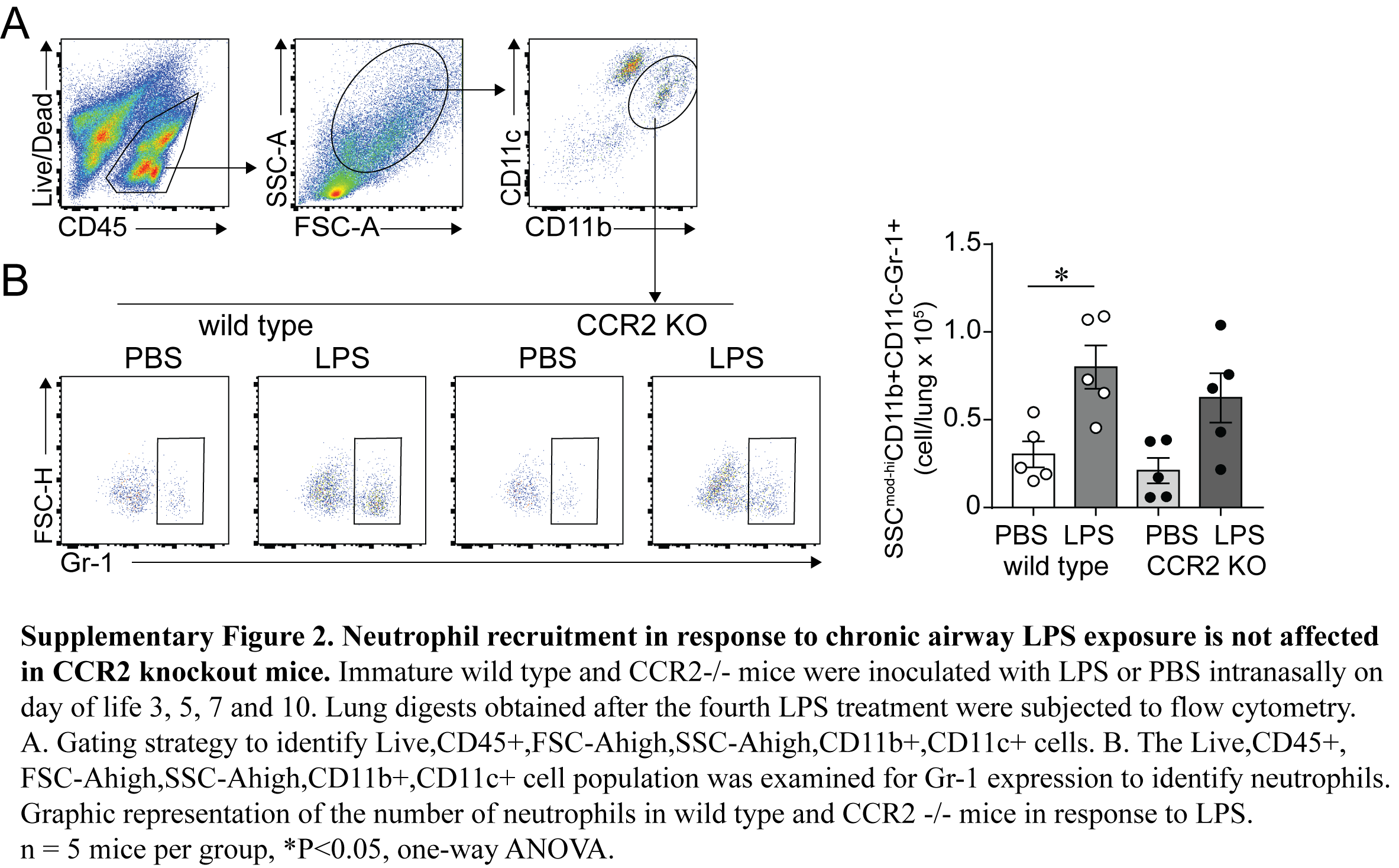

Supplement: Supplementary file 2 [file Image_2.tif]
